# Supplementary material for: Discontinuous rate-stiffening in a granular composite modeled after cornstarch and water
Source: Nat Commun. 2019 Mar 25;10:1283. doi: 10.1038/s41467-019-09300-z (PMC6434057; doi:10.1038/s41467-019-09300-z)
Supplement: Supplementary file 1 — Supplementary Information [file 41467_2019_9300_MOESM1_ESM.pdf]

Supplementary Information

Discontinuous rate-stiffening in a granular composite modeled after cornstarch and water

Chen *et al*.

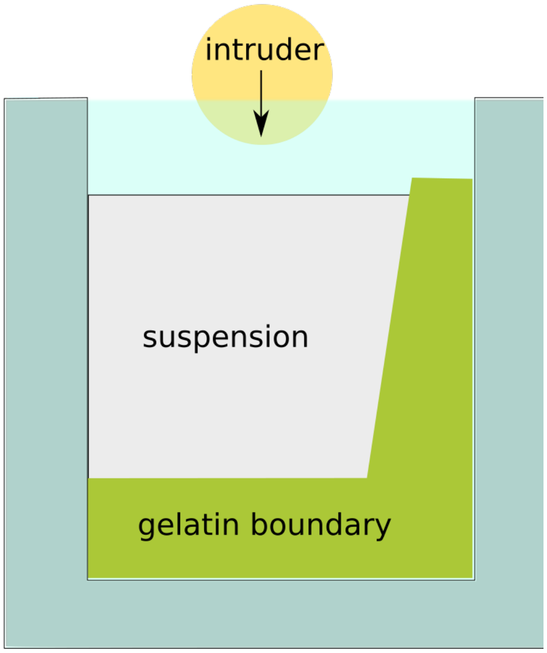

Supplementary Figure 1: Diagram of the suspension intruder setup with photoelastic gelatin boundary.

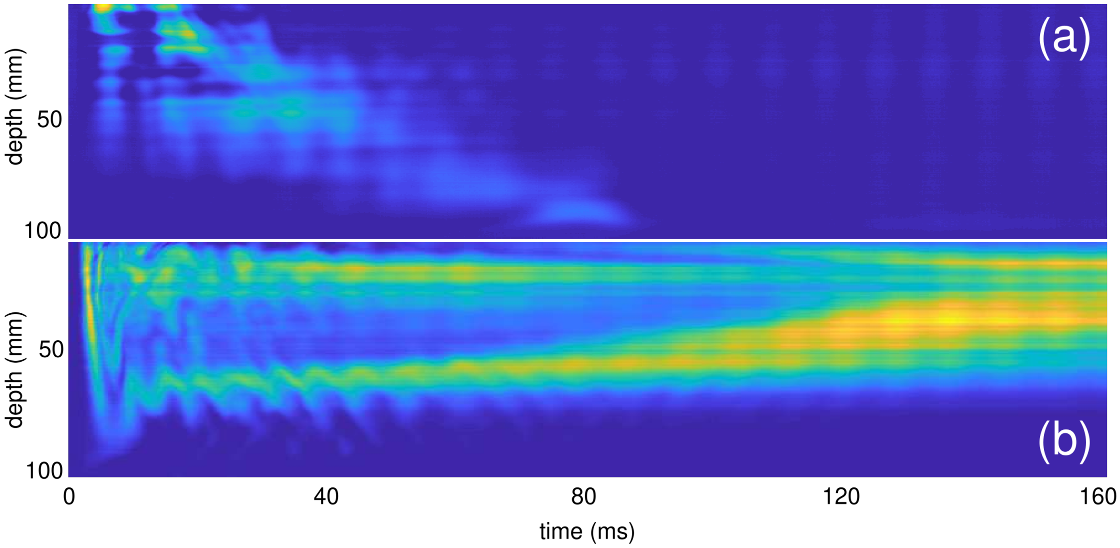

Supplementary Figure 2: Propagation of the pressure wave through the boundary. Space-time plot of photoelastic signals from the edge of the suspension after impact in a) cornstarch and ethanol, and b) cornstarch and water. Blue corresponds to qualitatively low force intensity, and yellow to high force intensity.

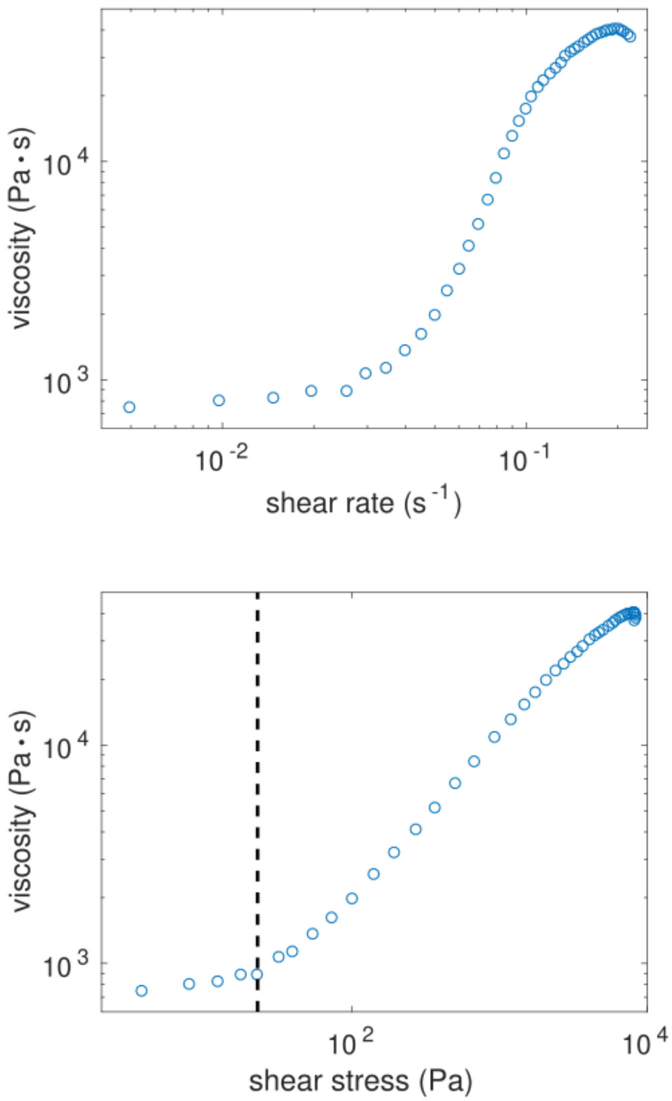

Supplementary Figure 3: Rheometry experiments on cornstarch and water. The viscosity is plotted vs shear rate (upper figure) and shear stress (lower figure). In the lower figure, the dashed line indicates the critical shear stress (force scale), ~20 Pa, needed for DST to happen. The data was collected with *TA Instruments AR G2 Rheometer*.

Supplementary Table 1: Experimental data on measurements of the total volume of cornstarch and water mixture.

Cornstarch Mass (g)	Water Mass (g)	Cornstarch Volume before Swelling (mL)	Water Volume (mL)	Measured Total Volume (mL)
200.22	301.91	125.92	301.91	440
188.76	200	118.72	200	335
242.04	204.64	152.23	204.64	371

Supplementary Note 1: Impact on cornstarch suspensions

We dropped a metal disc from varying heights into a cornstarch suspension (water and ethanol) with packing fractions φ = 45% (the packing fraction is calculated with density is 1.59 g cm^-3, without accounting for swelling). The suspension was enclosed in a rectangular acrylic channel ($h \times l \times w$ = 177×138×15 mm), with 35% occupied by the gelatin boundary, whose shape is shown in Supplementary Figure 1. The disk was guided by a chute located above the container. The disk had a diameter of 63.5mm, and width 11mm, mass 291g. We recorded impacts with a Photron FAST- CAM SA5. We tracked the impactor using a circular Hough transform at each video frame then numerically computed the velocity. Supplementary Figure 2 shows the photoelastic signal at the gelatin boundary after impact in cornstarch in ethanol and cornstarch in water.

Supplementary Note 2: Calculation of the actual cornstarch density in the cornstarch and water mixture

There are two parts we need to take into account in our calculation: cornstarch swelling, which increases the cornstarch volume, and water absorption in cornstarch, which increase the total mass in cornstarch. For a given cornstarch volume V_c, the actual volume after swelling is $1.4V_c$ (12% increase in diameter). Assume the volume of water absorbed in cornstarch is $a*V_c$. Then from the volume measurement of cornstarch and water mixture mentioned above, we have the total volume V_t expressed in two ways: $V_t = 1.11V_c + V_w = 1.4V_c + (V_w - a*V_c)$. This gives a = 0.29. Hence, the actual cornstarch density in the cornstarch and water mixture is: $\rho = (\rho_c*V_c + \rho_w*a*V_c)/(1.4*V_c) = (1.59 + 0.29)/1.4 = 1.34$ g cm^-3.
