# Supplementary material for: Discontinuous rate-stiffening in a granular composite modeled after cornstarch and water
Source: Nat Commun. 2019 Mar 25;10:1283. doi: 10.1038/s41467-019-09300-z (PMC6434057; doi:10.1038/s41467-019-09300-z)
Supplement: Supplementary file 3 — Description of Additional Supplementary Files [file 41467_2019_9300_MOESM3_ESM.pdf]

Description of Additional Supplementary Files

File Name: Supplementary Movie 1
Description: *Cornstarch swelling*: Video showing variation of cornstarch grain sizes under microscope as water evaporates.

File Name: Supplementary Movie 2
Description: *Heat transfer measurement during mixing cornstarch and water*: Measurement of heat transfer during mixing cornstarch and water. Heat transfer is measured with a thermometer immersed in water while adding and mixing cornstarch. As comparison, another thermometer is immersed in water with no cornstarch, showing no change of water temperature.

File Name: Supplementary Movie 3
Description: *Heat transfer measurement during mixing cornstarch and ethanol*: Measurement of heat transfer during mixing cornstarch and ethanol. Heat transfer is measured with a thermometer immersed in ethanol while adding and mixing cornstarch. As comparison, another thermometer is immersed in ethanol with no cornstarch, showing no change of ethanol temperature.

File Name: Supplementary Movie 4
Description: *Impact into cornstarch water and cornstarch ethanol suspensions*: Impact into cornstarch water suspension (left) and cornstarch ethanol suspension (right). Suspensions are contained with boundaries, two of which (right and bottom) are made of photoelastic materials (gelatin). Intensity variation of these two boundaries during the impact indicate stress variation and propagation in boundaries from the suspension.

File Name: Supplementary Movie 5
Description: *Impact into cornstarch+water and cornstarch+ethanol suspensions*: Impact into bare sand (left) and PDMS-coated sand (right).

File Name: Supplementary Data 1
Description: *Cornstarch particle size measurement without water*: Size measurement of dry cornstarch particles under microscope. Values shown in the figure are in the unit of pixel.

File Name: Supplementary Data 2
Description: *Cornstarch particle size measurement in water*: Size measurement of cornstarch particles immersed in water under microscope. Values shown in the figure are in the unit of pixel.

File Name: Source Data – Suppl. Fig. 2a
Description: Source data needed for reproducing Supplementary Figure 2(a) in the Supplementary Information. The row number corresponds to depth in pixel and the column number corresponds to time serie.

File Name: Source Data – Suppl. Fig. 2b
Description: Source data needed for reproducing Supplementary Figure 2(b) in the Supplementary Information. The row number corresponds to depth in pixel and the column number corresponds to time serie.

File Name: Source Data – Digs. 1-4 and Suppl. Fig. 3

Description: Source data needed for reproducing Figs. 1-4 in the main manuscript and Supplementary Figure 3 in the Supplementary Information.
